# Human Bone Marrow Subpopulations Sustain Human Islet Function and Viability *In vitro*

**DOI:** 10.9734/BJMMR/2015/17536

**Published:** 2015-05-18

**Authors:** Lu Guang Luo, Fang Xiong, Philippe Ravassard, John ZQ Luo

**Affiliations:** 1 Department of Medicine/Research, Roger Williams Medical Center, Boston University, USA; 2 Department of Molecular Biology, ICM, Biotechnology & Biotherapy Group 47 Bd de Hospital, Paris France; 3 Department of Medicine, Brown University, Alpert Medical School, Providence, Rhode Island, USA

**Keywords:** Allogeneic bone marrow, mesenchymal cells, endothelia cells, human Islet, β-cell regeneration

## Abstract

**Aims:**

Allogeneic bone marrow (BM) has been shown to support human islet survival and function in long-term culture by initiating human islet vascularization and β-cell regeneration. Various BM subpopulations may play different roles in human islet functions and survival. In this paper we investigated the effects of BM and its subpopulations, endothelial progenitor cells (E) and mesenchymal (M) cells on human islet's β-cell function and regeneration.

**Study Design:**

Isolation and identification of subpopulations from human bone marrow and culture with allogeneic human islet to investigate effects of different cell population on human islet function and regeneration.

**Place and Duration of Study:**

Department of Medicine, Center for Stem Cell & Diabetes Research, RWMC, Providence, RI, USA, between 2010 - 2014.

**Methodology:**

Human islets were distributed from Integrated Islet Distribution Program (IIDP) and human bone marrow (BM) was harvested by Bone marrow transplantation center at Roger Williams Hospital. BM subpopulation was identified cell surface markers through Fluorescence-activated cell sorting, applied in flow cytometry (FACS), islet function was evaluated by human ELISA kit and β cell regeneration was evaluated by three methods of Cre-Loxp cell tracing, β cell sorting and RT-PCR for gene expression.

**Results:**

Four different BM and seven different islet donates contributed human tissues. We observed islet β-cell having self regeneration capability in short term culture (3∼5 days) using a Cre-Loxp cell tracing. BM and its subtype E, M have similar benefits on β cell function during co-culture with human islet comparison to islet only. However, only whole BM enables to sustain the capability of islet β-cell self regeneration resulting in increasing β cell population while single E and M individual do not significantly affect on that. Mechanism approach to explore β-cell self regeneration by evaluating transcription factor expressions, we found that BM significantly increases the activations of β-cell regeneration relative transcription factors, the LIM homeodomain protein (Isl1), homologue to zebrafish somite MAF1 (MAFa), the NK-homeodomain factor 6.1 (NKX6.1), the paired box family factors 6 (PAX6), insulin promoter factor 1 (IPF1) and kinesin family member 4A (KIF4a).

**Conclusion:**

These results suggest that BM and its derived M and E cells enable to support human islet β-cell function. However, only BM can sustain the capability of β-cell self regeneration through initiating β-cell transcriptional factors but not individual E and M cells suggesting pure E and M cells less supportive for islet long-term survival *in vitro*.

## 1. Introduction

Islet β cell lost function after islet transplantation hinders successful islet transplantation [1,2]. Isolation process damage islet may result in β-cell lost function and caused apoptosis *in vivo*. Generating new β-cells from stem cells may be a solution from promising exploring researches, such as the differentiation of insulin producing cells from embryonic and adult stem cells [3,4] as well as the production of induced pluripotent stem (iPS) cells [5]. Valuable adult stem cell resource, such as adult bone marrow (BM) derived stem cells, has been considered the best-characterized stem cell source. BM and its derived stem cells have been used in the repair of heart [6,7], neuron [8,9] and muscle [10] injury. BM can support pancreatic growth *in vivo* and could potentially be manipulated to differentiate into β cells *in vitro* [11]. BM-derived stem cells migrate towards damaged islet site and differentiate into β cells under the influence of factors from the microenvironment (e.g. cell-cell, cell-extracellular matrix interactions and growth factors) [12,13].

Results from several studies demonstrate that adult BM cells are able to regenerate pancreatic cells through both neogenesis and transdifferentiation with no loss of function *in vivo* [14]. However, much debate surrounds the derivation of insulin-producing cells from BM cells [15] because generated cells lack important characteristics of normal β cells, and much more information about the several possible mechanisms of regeneration is necessary. BM has been found to be involved in pancreatic islet development during the neonatal period and after pancreatic injury [16]; cytokine treatment facilitates BM differentiation into β cells *in vivo* [13]. BM co-cultured directly with human islets repair isolation-induced injury in pancreatic islets and to prolong islet β cells viability [17]. We hypothesize that injured human islet from the isolation process may be repairable by BM cells and utilizing BM will be greatly improving human islet β cell survival resulting in successful islet transplantation for diabetic therapy. Friedenstein et al. provided the earliest evidence that adult bone marrow contains endothelial stem cells (E) and primitive mesenchymal stem cells (M) [18], and it is now well documented that M has the ability to differentiate into multiple lineages including osteogenic [19], adipogenic [20] and chondrogenic tissues [21]. Various BM subpopulations may play different roles, such as BM-derived E through angiogenesis and vascularization, while M creates a biological scaffold microenvironment with stromal cell paracrine function. It is crucial in preventing islet loss during transplantation by developing interventions that reduce or prevent injury-induced apoptosis or necrosis resulting in islet immunoreactions and β cells functional impairment [22]. Several mechanisms enable donor M to evade host allogeneic responses [23]. Endothelial progenitor cells (E), another special BM derived population present in BM throughout life, play a role in blood vessel formation in various tissues. In a study of mice subjected to inject E cells into injured pancreas, the number of both donor and recipient E cells increased after transplantation, but no E cells differentiation into β cells was found [24]. Identification of biological role of various BM derived subpopulation in human islet function and survival is critical importance for stem cell and β cell biology.

We have previously reported that allogeneic bone marrow supports human islet survival and function for more than six months [17] and islet tissue may in effect be regenerated through a synergy of angiogenesis and paracrine mechanisms. Bone marrow reduced the release of IL-1β in islets, thus inhibiting the apoptotic process in cultured islets [25,26]. Allogeneic BM derived M and E may individually create suitable islet microenvironment through E initiating angiogenesis for the revascularization of islets to repair destroyed microvessels and M through its paracrine activation to support islet function [27]. M and E could synergistically form human islet vascularization [28] and mediate paracrine function through releasing cytokines and growth factors, which turn on β cell regeneration genes and stimulate islet cell division.

In this study, we focused on identifying the biological effects of BM and its derived subpopulations on human islet function and regeneration. We also approach potential mechanisms by determining regeneration relative transcription factor activations for β-cell regeneration. Since human islets differ significantly in morphology from islet in other species [29,30], utilizing human tissue is the best approach to examine the biological effects and the mechanisms of BM on islet β cell function and survival process, which are frequently confounding factors in human islet biology in diabetic studies.

## 2. Materials and Methods

### 2.1 Tissue and Cell Culture

#### Human pancreatic islets

Human islet tissue (normal donors) was obtained from Islet Resource Centers (Integrated Islet Distribution Program, IIDP), Human Islet Laboratory at the University of Pennsylvania (Philadelphia, PA, USA), Massachusetts General Hospital (Boston, MA, USA), City of Hope National Medical Center (Duarte, CA, USA), University of Miami, School of Medicine, Diabetes Research Institute (Miami, FL, USA). Human islets were received from IIDP within 48 h after harvest from cadaveric donors. The viability of islets was >95% as determined by trypan blue dye exclusion after islets arrived. The Institutional Review Board (IRB) at Roger Williams Hospital and the IIDP Committees approved the use of these cells.

#### Human BM

Human BM (normal donors) was obtained under a separate Roger Williams Hospital IRB approved protocol. BM erythrocytes were eliminated by Ficoll-Paque™ Plus (Amersham Biosciences; Amersham, UK) per manufacturer directions. Cells were then washed twice with 10% Fetal Bovine Serum (FBS) in phosphate buffered saline (PBS), resuspended in culture medium (see below). Trypan blue (Gibco, 15250-061) staining was used to assess cell viability.

#### Allogeneic BM co-culture with human islets

Islets were placed in culture at a number of 50 islet equivalents (IEQs) per ml with 1×10^6^ allogeneic BM cells/ml. Cultures were maintained in RPMI 1640 (Gibco) supplemented with 10% heat inactived FBS (Hi-FBS, Hyclone), 5.5 mM glucose, 10 mM HEPES, and 1% P/S, pH7.3).

### 2.2 Human Islet Dissociation and Preparation

Human islets were washed with ice-cold PBS buffer and then 0.05% trypsin-EDTA with 0.2% or 0.6% collagenase was added. Cells were dissociated with sequential pipetting action after each period of 10-15 min incubation period at 37°C. At each step, islets were examined under light microscope to monitor the quantity of dissociated cells. To avoid cell death due to dissociation process, whole islets at each step were separated from the dissociated cells and transferred to a new small culture dish for further dissociation. After cell dissociation, trypsin and collagenase treatments were stopped with ice-cold PBS containing 5% FBS. The cell suspension was then passed through a cell strainer (40 micron, Becton-Dickinson, San Jose, CA) to remove debris and clumps, and resuspended in 5% HiFBS PBS.

### 2.3 Identification of MSC and EDC

CD 73 and 90 markers were selected using FACS for Human Bone Marrow Mesenchymal Cell Identification. PE mouse anti-human cd73 (BD Bioscience, cat.: 550257, 100tests) and FITC Mouse Anti-human CD90 (BD Bioscience, Cat: 555595) were used to identify cultured human MSC and cd31, cd73, cd135 and cd34 (BD Bioscience) were used for endothelia cells identification by FACS.

### 2.4 Enzyme-Linked Immunosorbent Assay (ELISA) for Insulin Measurement

#### Human insulin assay

Insulin concentrations in each specimen (culture medium and cell extracts) were measured using Ultra Sensitive Human Insulin ELISA Kit (Milllipore, EZHI-14K) according to the vendor's instructions and insulin concentrations were calculated using KC Junior^®^ microplate reader software (Bio-Tek Instruments, Inc.) [31]. Briefly, insulin standards and appropriately diluted samples were added to an insulin antibody-coated 96-well microplate and, incubated for 1 hour at room temperature after adding the detection antibody. After washing five times, anti-human insulin enzyme conjugate was added to the well and incubated for 30 min at room temperature. After washing five times, an enzyme substrate solution was added and then incubated 10 to 15 min at RT in the dark. The reaction was halted by adding 1 N sulfuric acid. Absorbance at 450 nm was read with a μQuant microplate reader (Bio-Tek Instruments, Inc., Winooski, VT) and concentrations were calculated by KC Junior® microplate reader software (Bio-Tek Instruments, Inc.) [17].

### 2.5 Quantification of β-cells Using Two-Step Intracellular Anti-insulin Staining/Flow Cytometry

Single β cells were washed and then resuspended in PBS for cell counting. The cell viability was measured with trypan blue. Cells were fixed in 4% paraformaldehyde solution for 20 min in the dark at 4°C with slight shaking every 5 min. Cells were then washed in ice-cold PBS and permeabilized in 0.05% triton x-100 solution/PBS for 15 min. Cells were washed and resuspended with ice-cold PBS buffer (including 5% HiFBS). 100 μL of cell suspension was added to each round-bottom polystyrene tube for the antibody staining. Cells were stained with antibody to Insulin or proinsulin for 30 min on ice. After incubation, cells were washed with and resuspended in ice-cold FBS buffer. Cells were then stained with the secondary antibody conjugated with fluorescein isothiocyanate (FITC) for 30 min on ice and in the dark. Lastly, cells were washed by centrifugation at 300g for 10 min and resuspended in 400 μL FBS buffer. Flow cytometry analysis was performed on Becton Dickinson LSRII system (BD Biosciences, CA, USA). In all cases, more than 10,000 cellular events were analyzed per tube, and in several cases, 100,000 cellular events were analyzed per tube.

### 2.6 Identifying the Source of Insulin Positive Cells Via Cre-LoxP

A cell-lineage tracing method was used to determine whether BM-derived stem cells have the potential to differentiate into functional islet cells. Two vectors were constructed, one expressing Cre recombinase under control of the insulin promoter (RIP-Cre) and the other a reporter cassette with the structure CMV promoter-loxP-DeRed2-LoxP-eGFP. The latter vector expressed the fluorescent marker DsRed2 (red) in all cells infected, while expression of eGFP is blocked. Removal of the DsRed2 coding sequence between the two loxP sites in beta cells infected by both vectors eliminates DsRed2 expression in these cells and activates eGFP expression. Non-β cells infected with both vectors are expected to express only DsRed2. We transfected BM and islets separately with two targeting vectors (TVs) before coculture, and we cultured transfected BM with untransfected islets. Results were evaluated under fluorescent microscopy and cell counts for GFP positive cells during coculture were conducted. We used 10^7^ PFU/mL-infected human islets, and about 60% of the cells were infected.

### 2.7 RT-PCR Qualification of Transcriptional Factor Activity

Total RNA was isolated from islet or islet and BM co-cultures using Trizol reagent (Invitrogen, catalog no# 10296-028). Isolated RNA was treated with Turbo DNA-free (Ambion, Catalog no# AM1907) to remove genomic DNA cDNA was synthesized from 500ng of total RNA using Applied Biosystems TaqMan® reverse transcription reagents kit (Applied Biosystems, catalog no.: N808-0234) with random hexamers in a total volume of 100 μl according to the manufacturer's instructions. cDNA was stored at -20°C for downstream PCR. Quantitative real time PCR was performed using Superarray SYBR green PCR kit (Superarray, Catalog no.: PA-012) in a total volume of 25 μl on Applied Biosystems 7000 sequence detection system (Applied Biosystems). The signal levels were normalized with GAPD for quantitative real-time PCR. Conditions for the PCR reaction were 10 min at 95°C, and then 40 cycles each consisting of 15s at 95°C, 60s at 60°C.

### 2.8 Statistical Analysis

Results are displayed as mean ± standard deviation (SD). Statistical significance was determined using the Student's t test; *p* values less than 0.05 are considered statistically significant.

## 3. Results

### 3.1 Isolating and Identifying BM Subpopulation from Human BM

**Fig. 1:**
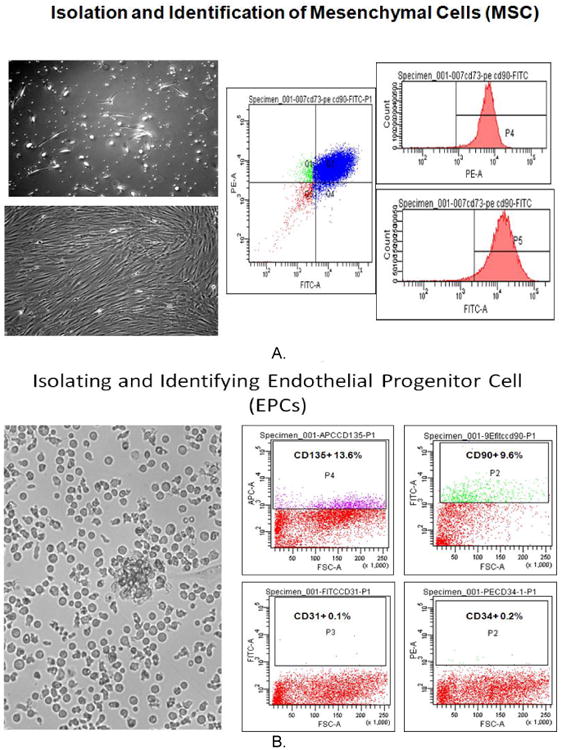
Isolation and identification of E (EPCs) and M (MSCs) Attached M in culture (Fig. 1 A left up image day 1) after one week culture (Fig. 1 A left bottom day 7) were scribed by rubber scriber and labeled with cell surface maker CD-73 and 90 after flow cytometry analysis (Fig. 1 A right panels) shows more than 95% cells are CD-73 and 90 positive cells. Cultured one week E cells (Fig. 1 B left image) were released and labeled with cell surface markers CD-135, 90, 31 and 34. Flow cytometry analysis indicated that these cells include most of positive CD-135, 90 and 31 but bare 34 positive. (Fig. 1 B right panels)

Bone marrow mesenchymal cell isolation and identification: Briefly, after three days of culture, floating cells were aspirated leaving only the attached cells for continued culture which include most of mesenchymal cells (M). Culture for three days, detached cells from attached culture and cells were labeled with CD73 and CD90 antibodies and identified by flow cytometry. The results indicated that >95% cells were either CD73 or CD90 positive suggesting that these isolated cells from BM were M. Using same technique, we characterize cell populations from cultures with CD31, CD73, CD135 and CD 34 antibodies and identified by flow cytometry. The results indicated that > 14% cells were CD31, CD73, CD135 positive but less than 1.5% was CD34 positive, suggesting that these isolated cells from BM were endothelial cells (E) (Fig. 1).

### 3.2 Effects of M, E and Mixture Whole BM (mwBM) on Human islet Function as Evaluated by ELISA for Human Insulin Release

**Fig. 2:**
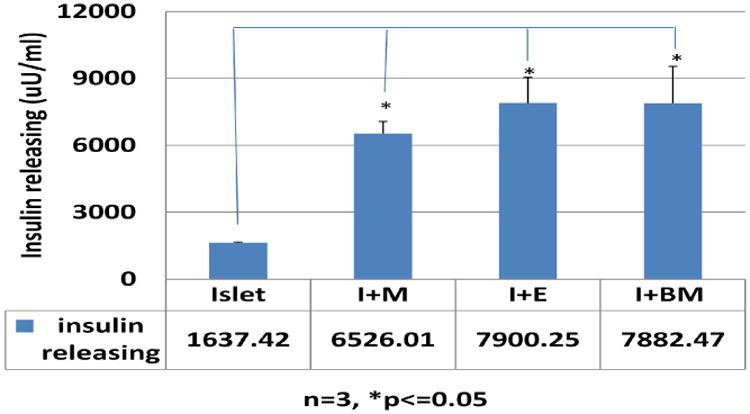
Evaluation effects of E, M and mwBM on human islet β cell function After 4 weeks culture, human islet β-cell function was evaluated by human insulin ELISA assay. the results indicated that all three cell populations in culture promotes human islet β-cell function vs. islet only culture 1637.42 μU/ml) (P<0.01, N = 3). however, no significant difference of M, E was found along with mwBM groups (6526.01, 7900.25 and 7882.47μU/ml)

Using the same culture protocol for BM/human islet co-culture, three different subpopulations, mwBM, purified M and E, from same bone marrow donor were co-cultured with human islets from same donor. Co-cultured tissue medium was collected on day 30. Human islet insulin release in medium was assayed by human insulin kit and results show that co-culture averaged insulin release no statistically significant difference amongst the three groups: mwBM (7882.47±1647.17 μU/ml), E (7900.25±1149.27 μU/ml) and M (6526.01±542.42 μU/ml). However, all three groups showed statistically elevated levels of insulin when compared to the islet only group (1637.42±33.35 μU/ml) p< 0.01 n = 18. (Fig. 2).

### 3.3 Different Effects of M, E and mwBM on Human islet β-cell Population as Quantified by Flow Cytometry and Cre-Loxp cell Tracing Technologies

**Fig. 3:**
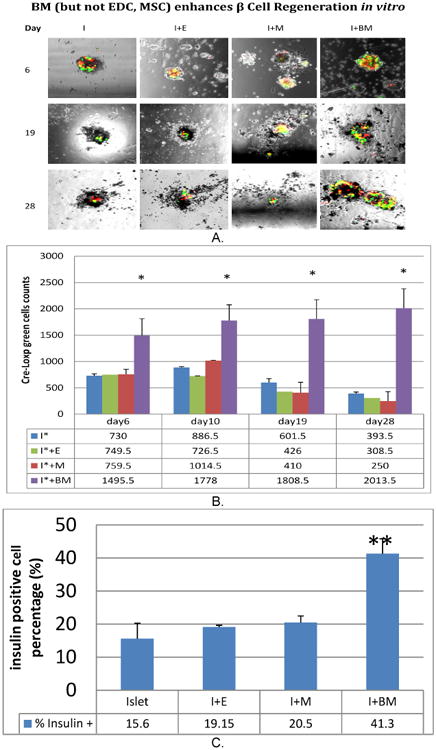
mwBM enhances β-cell regeneration *in vitro* but not E and M Cultured human islet cells labeled by Cre-loxp with mwBM, E and M were monitored under microscope with quantification of accounting green positive cells during culture days of 6, 10, 19 and 28 (Fig. 3 A). Top image panels show four groups Cre-Loxp positive cells in human islet culture, bottom panel indicates cre-loxp green cells quantification. Flow cytometry quantification method to quantify total positive insulin cells change after 28 days culture with different BM cell populations (Fig. 3 B)

During the cultures, effects of M, E and mwBM on human islet β-cell population was monitored by counting labeled human islet with Cre-Loxp on Days 6, 10, 19 and 28. As shown by previous data (Fig. 3), no significant differences in insulin positive cell population by cell counting represents amongst the four groups of culture in the first four days. MwBM co-culture showed a significant increase in insulin positive cells until day 28 (p< 0.01). M and E had no significant effects in terms of increase counting insulin positive cells (P< 0.05) (Figs. 3 a, b). Utilizing cell quantification via a two-step Flow Cytometry method to further quantify the total number of insulin positive cells, the results were consistent with Cre-Loxp studies. MwBM significantly increased human islet β-cell numbers throughout 4 weeks of culture. This effect was not seen in M and E cocultured islet (Fig. 3 c).

### 3.4 Mechanism Approach to Evaluate Effects of M, E and mwBM on Human Islet Regeneration Relative Transcription Factor Activations by Quantitative RT-PCR Evaluation

**Fig. 4:**
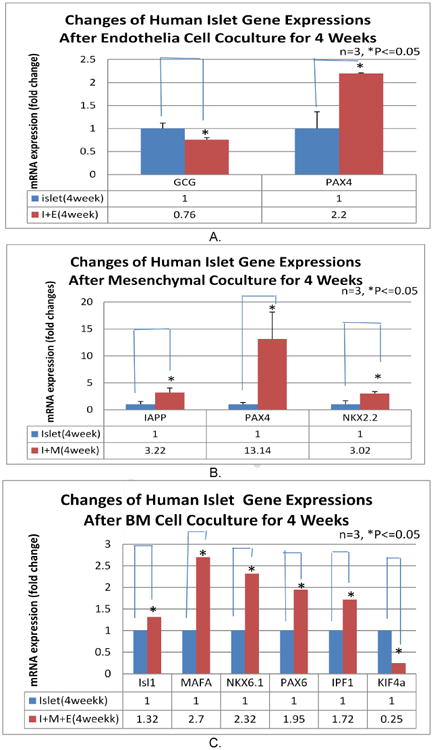
Difference of gene expressions in four different BM cell populations in human islets Isolated RNA from human islet tissue after culture 4 weeks was evaluated for transcriptional factors activation by RT-PCR. The changes show to comparison to islet only group. Only two factor activations were detected in EDCs group (A), three in MSCs group (B) and six in BM group (C). (* = p<0.05 n = 3)

A total of 24 β-cell differentiation and insulin related genes were evaluated and analyzed on culture day 28. The results showed that mwBM co-cultured islets induced the activation of six factors: Isl1, MAFA, Nkx6.1, PAX6, IPF1 and down-regulated Kif4a *vs*. islet only culture (p<0.05, Fig. 4). However, E cocultured islet induced two factor expressions Glucagon (GCG) and the paired box family factors 4 (PAX4) *vs.* islet only culture. M cocultured islet induced three positive activations: islet amyloid polypeptide (IAPP), PAX4 and the NK-homeodomain factor 2 (NKx.2) *vs.* islet only culture (p< 0.05). (Reference to these factors: Pandian GN, Taniguchi J, Sugiyama H. Cellular reprogramming for pancreatic β-cell regeneration: clinical potential of small moleculae control. Clin Tansl Medicine 2014, 3:6).

## 4. Discussions

Previous studies have revealed that human allogeneic BM has capability to maintain long-term human islet β-cells survival and function [17] and angiogenesis [32]. Human islet β cells replace cell loss through regeneration as a primary mechanism and lost regeneration capability results lost function shortly *in vivo* and *in vitro*. To identify whether a single BM derived subpopulation has the same effects as mixture whole BM, we examined the role of mesenchymal and endothelial cells, two major subpopulations of BM, on human islet β-cell function and regeneration. We found that M and E cells improved human islet function through increased insulin secretion (Fig. 2), however, neither populations display a significant effect on human islet β-cell regeneration as seen in mixture whole BM co-culture. The mesenchymal, endothelial cells and mixture whole BM were from same donor and human islet also were from same resource under the similar culture conditions, the significant difference of β cell population from culture groups is highly likely due to the characteristics of the different BM subpopulations versus a difference in tissue sources (BM and islets).

The effects of BM on human islet function observed in this study are consistent with most of reported results [20,33]. However, pure M and E cells improved islet β-cell function may be through mechanisms such as initiating angiogenesis [34] and preventing apoptosis [35] but less possibility of initiating β cells regeneration mechanisms supported by less significant β-cell regeneration relative transcription factor activations from human islet cocultured with M and E individually. Controversial reports on the effects of different subpopulations of BM on human islets may be a result of differing characteristics of sample sources across donors and species [36-39]. BM initiated islet β-cell regeneration relative gene expressions resulting islet β-cell regeneration suggests that BM sustaining the capability of human islet β cell regeneration in islet is the mechanism of BM increase coculture β cell populations. Other single and pure derived cell population (E and M) from BM in this study found less activation of β cell regeneration relative transcription factors resulting in no significant changes of human islet β cell populations. The results support that human islet sustaining β cell regeneration is critical for human islet survival and function in long-term. It can be explained that single pure cell from BM less effective to sustain human islet β cell function in long term culture because the pure population does not significantly sustain the capability of human islet β cell regeneration to replace islet β-cell loss during long term culture.

The results from this study support the concept that multiple BM cells increase the number of β-cell population through sustaining human islet β-cell regeneration (Figs. 3, 4) resulting in human islet function and survival in long term culture. However, BM derived subpopulations supporting β-cell function could be independent from the β cell self regeneration as seen by co-culture with BM (Fig. 2). This effect could be due to repair islet injure and improve cellular insulin secretion activation (limited preliminary studies not included here).

## 5. Conclusion

We can conclude that 1) Human BM and its derived subpopulations M and E repair islet injury, improve and expand β-cell function; 2) BM sustain human islet longevity through initiating β cell regeneration relative transcription factor activations to sustain human islet regeneration capability resulting β cell population increase; 3) Endothelial progenitor and mesenchymal cells improve β-cell function (through repairing islet injury) but less possible increase β-cell population because M and E to initiate less islet β cell relative transcription factors in our culture system.
